# Establishing a many-cytokine signature via multivariate anomaly detection

**DOI:** 10.1038/s41598-019-46097-9

**Published:** 2019-07-04

**Authors:** K. Dingle, A. Zimek, F. Azizieh, A. R. Ansari

**Affiliations:** 1 Centre for Applied Mathematics and Bioinformatics, Department of Mathematics and Natural Sciences, Gulf University for Science and Technology, P.O. Box 7207, Hawally, 32093 Kuwait; 2 Department of Mathematics and Computer Science, University of Southern Denmark, Campusvej 55, 5230 Odense M, Denmark

**Keywords:** Cytokines, Cytokines, Statistics, Statistics, Cytokines

## Abstract

Establishing a cytokine signature associated to some medical condition is an important task in immunology. Increasingly, large numbers of cytokines are used for signatures, via lists of reference ranges for each individual cytokine or ratios of cytokines. Here we argue that this common approach has weaknesses, especially when many different cytokines are analysed. Instead, we propose that establishing signatures can be framed as a multivariate anomaly detection problem, and hence exploit the many statistical methods available for this. In this framework, whether or not a given subject’s profile matches the cytokine signature of some condition is determined by whether or not the profile is typical of reference samples of that condition, as judged by an anomaly detection algorithm. We examine previously published cytokine data sets associated to pregnancy complications, brain tumours, and rheumatoid arthritis, as well as normal healthy control samples, and test the performance of a range of anomaly detection algorithms on these data, identifying the best performing methods. Finally, we suggest that this anomaly detection approach could be adopted more widely for general multi-biomarker signatures.

## Introduction

Cytokines are proteins involved in cell signalling and the immune system, and imbalances in cytokine levels are associated to many medical complications. Because of this, much research has gone into determining ‘cytokine signatures’ or profiles — typical multivariate cytokine concentration levels — of various groups, such as normal healthy populations, or cancer patients.

The benefits of establishing a cytokine signature are many, and include: Firstly, aiding diagnosis; for example, if a patient has some medical complication of unknown cause, one may wish to study their immune system to infer if any anomalous cytokine patterns are present, which may suggest that the immune system is playing a role in the complication (as opposed to purely physical trauma for example). In this case, it would be beneficial to have a reference signature for normal/healthy subjects to compare to^1,2^. Secondly, monitoring treatment of some medical complication which is known to correlate with cytokine imbalances, especially to detect if a course of medication is having any effect on the cytokines (e.g. in rheumatoid arthritis treatment^3^). Thirdly, in terms of basic science, a signature is of interest for a fundamental understanding of diseases and sickness, for instance, in developing potential treatments by complimenting or antagonising abnormal cytokine levels^4,5^.

Historically, measuring many different cytokines was both expensive and required substantial volumes of biological fluid. The advent of multiplex arrays has made simultaneously measuring tens and even hundreds of cytokines less expensive, and generally more feasible. Because of this, increasingly in the literature, many different cytokine concentrations are measured and used for deriving a signature. Some examples of many-cytokine signature studies are Azizieh *et al*.^1^ who studied a panel of 7 cytokines associated to different pregnancy complications; Haugen *et al*.^6^ who studied a panel of 27 cytokines in children with pneumonia; Yurkovetsky *et al*.^7^ who studied 29 cytokines from cancer patients; Pranzatelli *et al*.^8^ who studied 34 cytokines in cerebrospinal fluid from children with neurological disorders; Kleiner *et al*.^2^ who studied 48 cytokines from healthy subjects; Hosoki *et al*.^9^ who studied a panel of 48 cytokines from bronchoalveolar lavage fluids in asthma patients; and Nijaguna *et al*.^10^ who studied 48 cytokines to establish a signature for Glioblastomas brain tumours. Despite the fact that more cytokines can now be measured for a given sample of biological fluid, obtaining many human samples is still expensive and time consuming, which often results in datasets with small sample sizes. Multivariate data sets with relatively few samples pose challenges for statistical analysis and inference. Hence a low ratio of samples to variables is a common and challenging characteristic of cytokine data sets.

Currently, signatures are often specified via individual cytokine ranges, such as via min-max value ranges, boxplots (which use data quartiles), or other summary statistics (e.g.^2,8^), or analogous reference ranges for certain ratios of cytokines^11,12^. In this approach, a sample under investigation would be declared anomalous — i.e. not matching the cytokine signature — if one or some of the sample individual cytokine concentration values or ratios are abnormal, that is, outside of some given reference ranges. These individual ranges or ratios can be useful for identifying anomalous profiles, especially if one or a few cytokine values are very strongly abnormal, and they are also easy to interpret. However, given the fact that cytokines work in interacting networks, both promoting and suppressing each other, it may be better to use some method which can combine many cytokine values into one single score, which indicates whether or not a given subject’s multivariate profile is anomalous with respect to some signature (i.e. not typical of the signature). Moreover, this standard individual/ratio range based approach also suffers from a number of drawbacks, which we now describe in detail.

## Subjectivity and Expert Knowledge

Firstly, it will be challenging for a medical practitioner using individual ranges to fathom a list of tens or hundreds of cytokine ranges, and then mentally combine these to make a normal/abnormal classification judgment, for some given sample. Indeed, such a decision may be quite subjective, and require expert knowledge of cytokines and their balances. For example, it may not be clear whether a sample with 4 extreme values out of 48 cytokines should be classed as an anomalous sample, and if/how the decision will depend on which 4 cytokines they are, and how extreme they are. To make such decisions requires expert knowledge of cytokines, and indeed one expert may not agree with another. Ideally, a method would not be subjective or require expert knowledge, such that it could be implemented accurately, quickly and consistently across data for many patients. This problem would become even worse should the ratios and individual values be combined, leading to possibly hundreds or thousands of individual ranges and ratios to be taken into account by the practitioner.

## High-Dimensional Data

Secondly, the problems associated with having very-many variables is well studied in anomaly detection^13^, and will affect many-cytokine anomaly analyses also. There are several reasons why many-variables cause difficulties, for instance, anomalous combinations of values can be ‘hidden’ by many irrelevant variables. Further, the probability that at least a few extreme values are observed in some variables becomes high, which may lead to *almost all* test samples being declared anomalous, even if the test samples are statistically identical to the reference samples (i.e. follow the same multivariate distribution). This can already be a problem if the number of variables (cytokines) is only ~10^13^, and is greatly exacerbated if the number of variables grows large, which is the case for current research where even hundreds^14^ of cytokines are analysed simultaneously. The problem of anomaly detection for many-variable data sets is even worse in the case of ratios: For *d* variables, there are $$(\begin{array}{c}d\\ 2\end{array})=d(d-\mathrm{1)/2}$$ ratios which grows large very quickly, even for modest *d*. When very many statistical tests are performed, such as testing “Is the ratio abnormal?”, there is a high probability that at least a few extreme ratios appear in the test samples, and hence that the sample may be declared anomalous. Hence the majority of any samples tested against the reference ranges may be declared anomalous, even if the test samples are statistically identical to the reference samples. Now, it is not common practice to test *all* pairs of ratios in cytokine studies to identify anomalous samples, but rather only ratios which are expected to be important (e.g. inflammatory vs. non-inflammatory). However, in the context of medical diagnosis where the origin of some symptom is unknown, the practitioner might not know *a priori* which cytokine ratios to investigate; indeed, even whether the symptom is related to cytokine values may be unknown. Therefore, ideally, most (or possibly all) ratio pairs would be investigated. Furthermore, even if prior knowledge guided a choice of some important ratios to investigate, with so many cytokines, even the set of ‘important ratios’ will grow large quickly, even if not as large as *d*(*d* − 1)/2. Fortunately, within statistics and especially *outlier analysis*^15,16^, the problem of identifying whether a sample is anomalous with respect to some large reference data set is well-studied, and *high-dimensional anomaly analysis*^13^ is the specialised study of difficulties arising with many-variable outlier detection, and approaches to overcoming them. The field of high-dimensional anomaly analysis has advanced significantly over the last decade or so, but as yet these advances have not (to our knowledge) been exploited in cytokine studies.

## Multimodal Distributions

Thirdly, multimodal cytokine distributions are not well handled by simple ranges, which may overlook low density ‘gaps’ in a distribution, and also strongly affect estimates of the standard deviation of the data, and typical ranges based on quartiles. For example, a value is commonly considered anomalous if it is outside of the range [*Q*_1_ − 1.5*IQR*, *Q*_3_ + 1.5*IQR*], where *Q*_1_ and *Q*_3_ are the first and third quartile of the reference data set, and *IQR* = *Q*_3_ − *Q*_1_. If the data is multimodal, then this range may be unrealistically stretched, due to a gap in the distribution between Q1 and Q3. Another common anomaly criterion is for a sample’s Z-score (i.e. the number of standard deviations from the mean) to be <−3 or >3. Even using this method, similar problems would arise from the bi-modal nature of the concentration values which would give misleading values for the mean and standard deviation of the distribution. Note that using ratios in place of individual values would not ameliorate this problem.

## Correlations

Fourthly, relying only on individual values will miss anomalies based on correlations, i.e. unusual combinations of cytokines. Looking for abnormal ratios of a pair of cytokine values (e.g. as in^11,12^) improves on just examining individual cytokine ranges, because some linear correlations between cytokines can be accounted for. Indeed, this method may be effective at anomaly detection in many cases, because cytokines often have roughly linear correlations (of logarithmic concentration values), for which ratios are a reasonable choice of metric. If any non-linear correlations exist between cytokines, then these may be missed. Having said that, nonlinear correlations are not known to be common for cytokines, and hence correlations are the least significant of these issues just detailed.

Here we suggest that what is needed in the task of developing a many-cytokine signature is a method which, given a large sample of cytokine measurements for members of some group (e.g. healthy children), and a single new subject to investigate (e.g. a sick child), will inform whether the new subject’s cytokine values are typical or not of the group. In other words, instead of comparing a single subject’s individual (or ratio) cytokine profile to reference ranges as a way to decide if their profile ‘matches’ the signature of some group of interest, we say a profile will ‘match’ some group signature if a multivariate algorithm classifies the profile as typical of reference samples for that group. On the other hand, the profile will ‘not match’ some group if the algorithm classifies the profile as anomalous, with respect to the group reference samples. In this manner, the cytokine signature is established via multivariate statistics and anomaly detection. To avoid potential confusion, we stress that the task at hand concerns comparing a single test profile (e.g. a sick child) to a group of reference samples (e.g. healthy children), and not comparing two groups of samples to each other, which is much more common in the medical statistics literature.

To illustrate our approach, we use several data sets, from pregnancy, to brain tumours, to rheumatoid arthritis, and normal healthy populations. To investigate whether the algorithms can appropriately handle the cytokine data sets, we test different algorithms from anomaly analysis, finding most to be very successful, suggesting that our proposed approach to making signatures is viable.

## Results

### Comparing multivariate algorithms

We will now test some multivariate anomaly detection algorithms on natural samples (i.e. experimentally measured) data. We experiment with six popular anomaly detection algorithms, namely: Correlation Outlier Probabilities (COP)^17^, Local Outlier Probability (LoOP)^18^, Local Outlier Factor (LOF)^19^, *k*-nearest neighbours (KNN)^20^, Global-Local Outlier Scores from Hierarchies (GLOSH)^21^, Isolation Forest (IsolFor)^22^, and Angle Based Outlier Detection (ABOD)^23^ - see Methods for more details of these algorithms. The algorithms return a score value for each sample in a data set, which indicates to what extent each sample is outlying, as compared to the other samples in the data. These scores then can be used to rank each sample, with higher ranks corresponding to more anomalous samples.

In order to measure which multivariate algorithm is best at distinguishing reference group samples (inliers) and test group samples (outliers), ideally we would have a reference data set and a set of samples which are known to be (non-trivial) outliers, and then compare algorithms by their ability to correctly classify inliers and outliers. If the algorithms correctly classify the inlier samples as more typical of the reference group samples, and correctly classify outlying samples as more untypical/anomalous with respect to the reference group, then the algorithm is deemed more successful and accurate. To quantify accuracy, we use the standard receiver operator characteristic area under the curve (ROC AUC) value, where an ROC AUC value of ~0.5 is very poor accuracy (no better than random guessing), and ~1 is close to perfect classification accuracy. An ROC AUC value can be interpreted as the probability that a random inlier and random outlier pair are correctly ranked as less and more likely to be an outlier, respectively. See the Supplementary Information for more ROC curve details and examples.

Finally, in the following, *n* denotes the number of inlier reference samples, and *d* the number of cytokines (i.e. the dimension of the data.).

### Normal delivery and hypertension (PIH-out)

The first data set we examine comes from the study of Azizieh *et al*.^1^ who analysed *d* = 7 cytokines from *n* = 53 women who had healthy pregnancies and normal deliveries (ND). These data will be the inlier reference data. As outliers, we will use data from the same study, where it was found that a subgroup of 9 women suffering pregnancy induced hypertension (PIH) had very different cytokine profiles (labelled PIH-out), as compared to the ND group. The PIH-out group will act as the test ‘outlier’ samples. We now study the question: Given the ND samples and a PIH-out sample, can the algorithms distinguish which samples are typical ND samples, and which is the outlier (PIH-group sample)?

**Figure 1:**
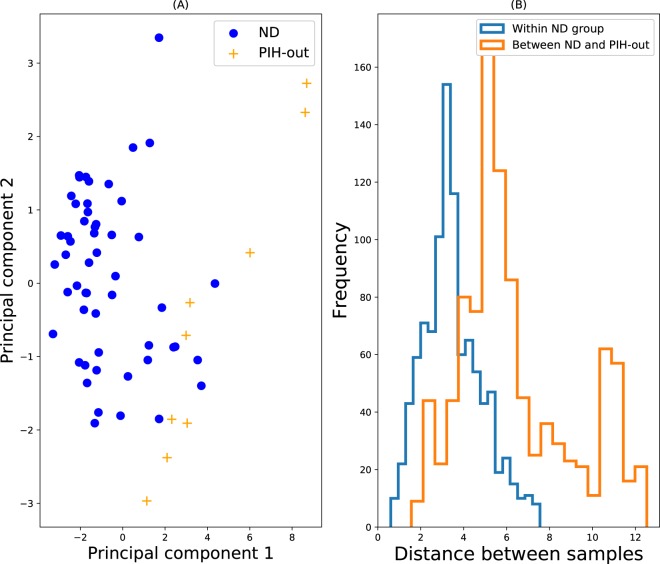
ND and PIH-out. (**A**) PCA plot. Blue circles represent the inlying reference data, and the yellow crosses denote the test data, which is assumed outlying. These plots help to visually appreciate what fraction of ‘outlier’ samples are in fact outlying, and to what degree they are outlying (if at all). (**B**) Distributions of pair-wise distances of both within the reference inlier group (blue), and between samples of the inlier and outlier group (orange). 1000 random pairs were chosen for each histogram.

Figure 1(A) shows a PCA plot of the data for the inlier and outlier group (the first two principal components account for 71% of the total variance). From the figure, we see that most PIH-out group samples appear far from the ND group, while a few samples are closer, suggesting that we may expect the algorithms to perform well and correctly rank inliers and outliers. In order to estimate the difficulty of the classification task at hand from a different perspective, we plotted the distribution of distances for random pairs of samples within the inlier group, and also for random inlier-outlier pairs between the two groups. If these distributions are almost indistinguishable, then this would suggest that the two groups are statistically very similar, whereas if the inlier-outlier pairs have larger distances typically, then we can infer that the outlier group contains samples which are strongly divergent from the inlier group, and hence should be detected as outliers by the algorithms. Figure 1(B) shows that for this ND vs. PIH-out data set, the difference in distances is quite pronounced. On the other hand, we have quite a small reference inlier data set of ~50 samples, and this poses a challenge for statistical anomaly detection. Considering these perspectives, correctly classifying the sample should be rather easy for the algorithms, but still a non-trivial achievement.

**Figure 2:**
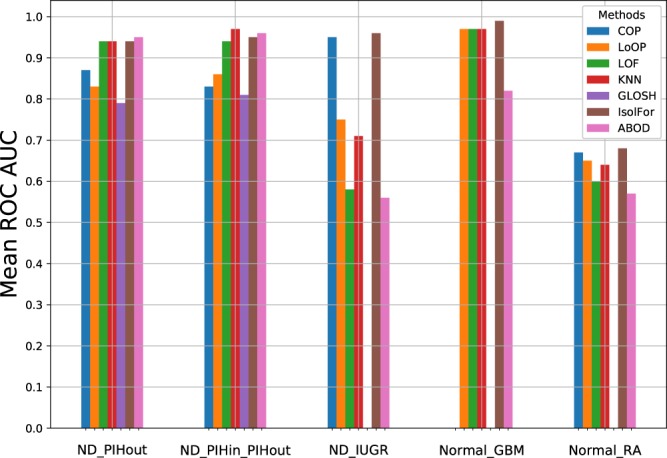
Comparing mean ROC AUC values for all algorithms, on all data sets. Means are calculated as the average ROC AUC values over 1000 subsamples of the outlier group (Methods). COP and GLOSH did not yield a ROC AUC result for some data sets, due to small data set sizes.

Consistent with the preceding discussions, Fig. 2 shows that the algorithms perform very well with this data set, achieving ROC AUC values from ~0.8 to ~0.95.

Before proceeding to the next data set example, we can illustrate some difficulties with the common individual/ratio range method. In this data set, we calculated that a large fraction (30%) of the ND data have at least one cytokine value which is outlying, based on the interquartiles criterion (described in the Introduction). This is not an artefact of the specific data set, but rather a natural consequence of the multivariate nature of the data. Indeed, theoretically, the higher the number of cytokines, the higher the fraction. That is, we expect one or a few ratios to be anomalously high or low, even for ND samples. Finding this large fraction is significant because it suggests that if individual cytokine values are used to detect unusual profiles, then there will be a high ‘false-alarm’ rate. As a consequence, if many of the ND samples themselves are deemed anomalous, then for a sample to be classified as anomalous becomes somewhat meaningless, and implies inaccuracies in classification. Turning to ratios, even with only *d* = 7 cytokines in these data, there are *d*(*d* − 1)/2 = 21 possible ratio pairs, and for this ND data set we find that *all* (100%) of the ND samples have at least one ratio pair which is outlying. So the false-alarm rate is even higher for ratios.

Using only individual values and ratios would be challenging because a medical practitioner would have to mentally combine a list of 7 individual values and up to 21 ratios typical of the ND samples, and another 7 individual value and 21 ratios for a subject under assessment, and then use subjective assessment and expert knowledge, confounded by the high false alarm rates just calculated, to form a judgement regarding whether the given subject is typical or not of the ND signature. Such a judgement is possible, but surely challenging, time consuming, and potentially inconsistent and inaccurate.

### Normal delivery and hypertension (PIH-in and PIH-out)

The next data set is same as the previous one from Azizieh *et al*.^1^, except that the inlier group will now consist of both the ND data above, and a subgroup (23 samples) of the PIH data, which Azizieh *et al*. found to be statistically almost indistinguishable from the ND samples, which they labelled PIH-in. Hence the test here for the algorithms is to correctly identify ND and PIH-in as inliers, and PIH-out as the outliers. For the combined data, *d* = 7 and *n* = 53 + 23 = 76.

**Figure 3:**
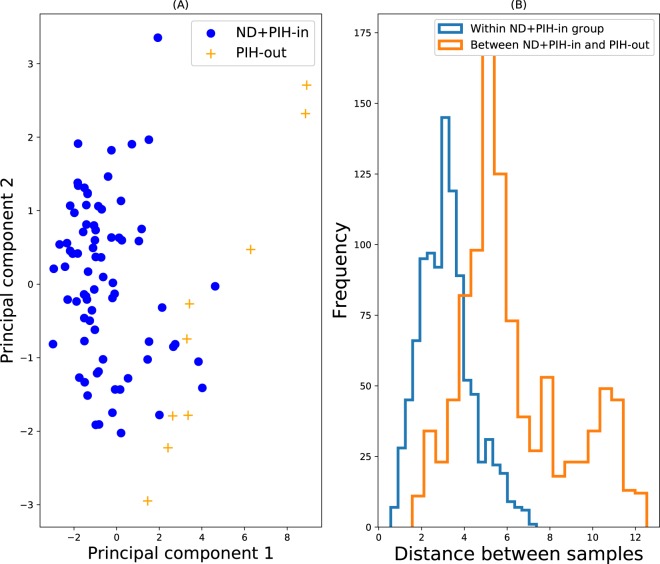
ND and PIH-in vs. PIH-out. (**A**) PCA plot. Blue circles represent the inlying reference data, and the yellow crosses denote the test data, which is assumed outlying. (**B**) Distributions of pair-wise distances of both within the reference inlier group (blue), and between samples of the inlier and outlier group (orange). 1000 random pairs were chosen for each histogram.

Figure 3(A) shows a PCA plot of the data for the inlier and outlier group (the first two principal components account for 70% of the total variance), and Fig. 3(B) shows a similar distance plot to the ND vs. PIH-out data. Again the algorithms perform very well, with Fig. 2 showing that all the algorithms achieve good ROC AUC values.

### Normal delivery and intrauterine growth restriction

Still in the context of pregnancy, but with a different data set consisting of different cytokines, we examine a sample of healthy normal delivery (ND) cytokine profiles, and cytokine samples from women presenting intrauterine growth restriction (IUGR). The data is from ref.^24^. There are 39 samples of IUGR, representing both symmetric, asymmetric and unknown forms, and these IUGR samples will form the outlier test samples. An additional challenge here is that there are more cytokines (*d* = 10) and fewer samples (*n* = 24) as compared to the previous data sets, and hence a very low ratio of samples to variables.

**Figure 4:**
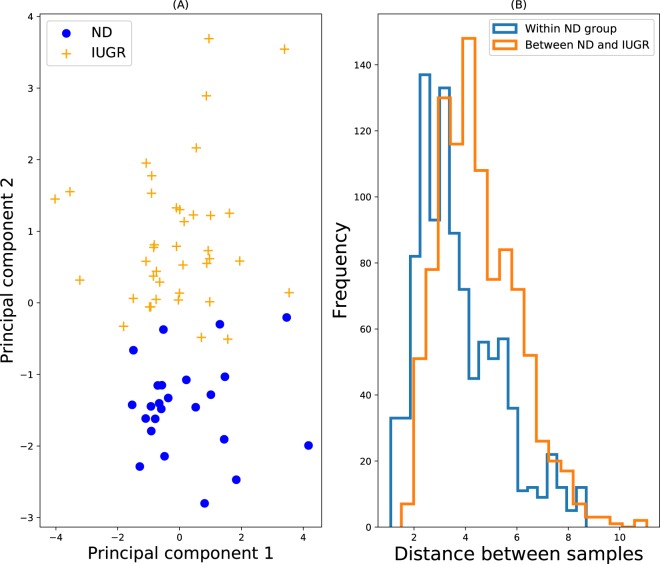
ND vs. IUGR (**A**) PCA plot. Blue circles represent the inlying reference data, and the yellow crosses denote the test data, which is assumed outlying. (**B**) Distributions of pair-wise distances of both within the reference inlier group (blue), and between samples of the inlier and outlier group (orange). 1000 random pairs were chosen for each histogram.

The PCA plot (accounting for 43% of variance) is shown in Fig. 4(A) and indicates that the two groups are clearly different, yet many IUGR samples appear close to the ND samples, and hence may not be detected as outliers. The distance distributions are not strongly different (Fig. 4(B)) also, and not many between-group samples have distances outside of what is typical of the intra-group ND distances. Again, this suggests that identifying the outliers may be hard. Turning to the computational experiments, Fig. 2 shows that Isolation Forest and COP performed very well, while many of the other algorithms struggled with this data set, due to the issues described above.

It is worth pointing out also that with 10 cytokines there are 45 possible ratios, and consistently 100% of the ND samples had at least one anomalous pair. Further, more than half (54%) of the ND samples had at least one individually outlying cytokine value with respect to (w.r.t.) the ND data.

### Normal controls and Glioblastoma brain tumours

Nijaguna *et al*.^10^ measured cytokine concentrations (from serum) of normal healthy people (*n* = 26 samples) and also from a gender and age matched group of 148 patients having Glioblastoma brain tumours (GBM). *d* = 48 different cytokines were measured.

**Figure 5:**
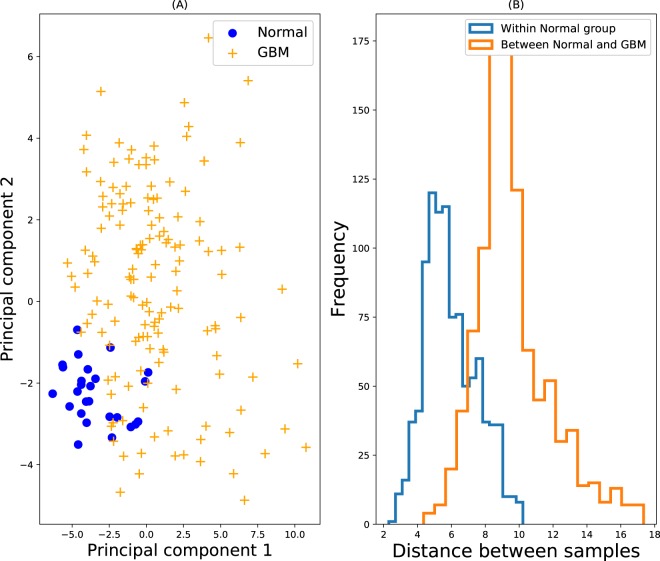
Normal vs. GBM. (**A**) PCA plot. Blue circles represent the inlying reference data, and the yellow crosses denote the test data, which is assumed outlying. (**B**) Distributions of pair-wise distances of both within the reference inlier group (blue), and between samples of the inlier and outlier group (orange). 1000 random pairs were chosen for each histogram.

Both Fig. 5(A,B) imply that the majority of GBM samples are anomalous w.r.t. the normal samples, even though some GBM samples are close to the normal samples. Hence, given a large sample of normal subjects’ cytokine values, we would expect a good algorithm to classify the majority of GBM samples as anomalous. With the current data set, we only have *n* = 26 healthy samples, which is very small, especially given the high-dimensional data set. Relatedly, this data set poses another challenge, which is that the number of reference samples for the inlier group is smaller than the number of variables (*n* < *d*), which is generally a challenge for statistical analysis. Given the small sample size, the ROC AUC values are very impressive, because Isolation Forest achieved a near-perfect score of 0.99, followed closely by KNN, LoOP and LOF. Note that both COP and GLOSH failed to yield ROC AUC values for this experiment, due to the very low ratio of samples to variables. Indeed, COP requires *n* > 3*d*, as a rule of thumb^25^.

**Figure 6:**
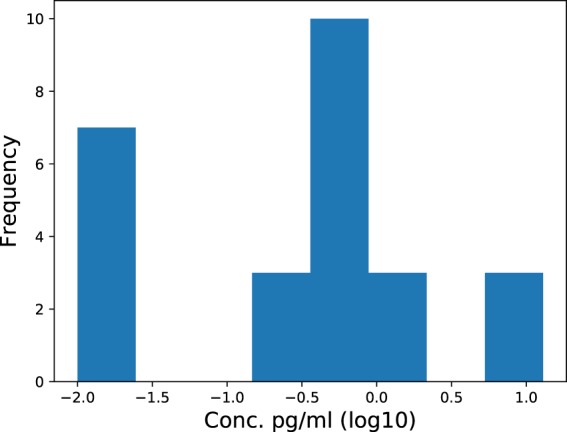
A histogram of IL-1A concentrations from the normal data. Some cytokine concentration distributions are bimodal, and for such cytokines, individual reference ranges based on quartiles can give misleading results regarding what concentration values are, and are not, abnormal.

For this 48-cytokine data set, the *majority* (89%) the normal samples have at least one cytokine value which is anomalous (extremely large or small) w.r.t. the normal samples, and 100% of the samples had at least one anomalous ratio out of the 1128 ratio pairs for this data set. Both of these results stem from the multivariate nature of the data, and both imply that using either individual or ratio values will be problematic for detecting outlying samples. The normal data also provides an example of the problem of multimodal data. Figure (6) shows a multimodal distribution for the cytokine IL-1A. If a typical reference range was given for this cytokine based on quartiles, the range for the log10 concentration value would be [−4.1, 2.3], which is quite misleading, because this interval does not account for the low density ‘gap’ in the distribution (i.e. from −1.6 to −0.8), and moreover the upper limit 2.3 is far beyond the upper limit of the distribution which is roughly 1.0. Hence samples with log10 concentration of 2.2 for example would be considered typical, even though it is apparent from the figure that such a value would be extreme.

### Normal and rheumatoid arthritis

**Figure 7:**
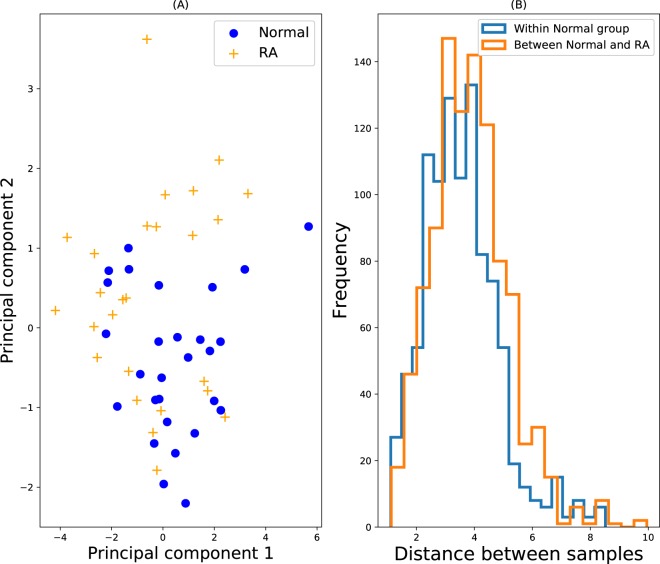
Normal vs. RA. (**A**) PCA plot. Blue circles represent the inlying reference data, and the yellow crosses denote the test data, which is assumed outlying. These plots help to visually appreciate what fraction of ‘outlier’ samples are in fact outlier, and to what degree they are outlying (if at all). (**B**) Distributions of pair-wise distances of both within the reference inlier group (blue), and between samples of the inlier and outlier group (orange). 1000 random pairs were chosen for each histogram.

For our final data set, the reference group consists of *n* = 28 normal/healthy subject samples of *d* = 8 cytokines, previously studied by Azizieh *et al*.^3^. The outlier group consists of 26 subjects suffering from rheumatoid arthritis (RA) of varying levels of severity. While Azizieh *et al*. found these two groups to be statistically different, examining Fig. 7(A) suggests that the groups overlap considerably and only few of the RA samples are so different to the normal data as to be considered outliers (principal components 1 and 2 account for 62% of the variance). Figure 7(B) also yields the same conclusion, with intra-reference group pairwise distances very similar to normal-RA group distances.

The fact that this data set has only few reference samples, and that the two groups appear similar, leads us to predict that the ROC AUC values will be low. Performing the calculations confirms the prediction, and Fig. 2 shows that all algorithms returned modest ROC AUC values, with the highest being Isolation Forest with a value of 0.68.

### Which algorithm performed best

We do not attempt here to compare algorithms in general for their performance, because comparative studies of the behaviour of multivariate algorithms for outlier detection are necessarily always restricted to the studied data-specific scenarios^26^. Hence we only make claims about the ability of the algorithms in the context of cytokine studies, whereas all algorithms will have advantages and disadvantages, depending on the data context.

Here we found the following average ROC AUC values (in brackets) across the data set examples: COP (0.83), LoOP (0.81), LOF (0.81), KNN (0.85), GLOSH (0.80), IsolFor (0.91), and ABOD (0.77). From this perspective, Isolation Forest (IsolFor) is the clear winner, and the other algorithms perform somewhat similarly to each other. It should be noted that Isolation Forest is in fact inherently an ensemble method and thus is theoretically expected to perform better than the other, individual methods^15,27^. Those could also be ensembled for improved performance^27^ which is, however, beyond the focus of this study.

Maximising the ROC AUC is not the only consideration; recall for example that COP and GLOSH failed to work when the number of samples *n* was too small relative to the number of variables *d*. So the number of samples and variables should also be taken into account when deciding which algorithm to use.

## Discussion and Conclusion

Deriving cytokine signatures from multiplex arrays is important for medicine and biology, especially in terms of diagnosis, monitoring disease, and a basic understanding of disease processes. Commonly, signatures are given as lists of reference ranges for each cytokine, or reference ratios of key pairs of cytokines. Here we have:

- Argued that standard approaches to creating cytokine signatures have important drawbacks, especially when the number of cytokines gets large, as is increasingly common in cytokine studies. Further, we have computationally illustrated some of these drawbacks, using example data sets;
- Proposed another way which signatures can be made via multivariate anomaly detection algorithms, which more accurately, objectively, and quantitatively classify a given cytokine profile of interest as typical or not of some given group;
- Tested various anomaly algorithms on natural data, and thereby shown that many of these algorithms accurately distinguish reference samples from example outlier samples, and Isolation Forest was found to be the most accurate for these cytokine data sets.

In this work we have focussed on whether a sample can be regarded as abnormal or not, and we have not considered the *explanation* for the outlier, that is, which variables contribute to the abnormal nature. In general, finding the explanation for why a high-dimensional sample is an outlier can be difficult, and many of the common algorithms do not provide such explanations directly. Hence this is another possible factor to consider when choosing an algorithm for a cytokine data set, because it is often interesting and medically important to know the outlier explanation. From among the algorithms we have studied here, COP is the only one (to our knowledge) which directly provides an explanation in terms of an “error vector”, indicating which variables are most unusual, which is a positive aspect of COP. With these considerations in mind, Isolation Forest could be a good algorithm for establishing a cytokine signature via anomaly detection, and if sufficient data (*n* > 3*d*) is available and outlier explanations are required, then COP could be used also, but nonetheless we recommend using several different methods simultaneously, to get a more complete picture.

Our cytokine signature approach also has drawbacks. One drawback is that the interpretation of abnormal profiles may not be so clear and easy to understand, which may be problematic for medical practitioners. For example, by comparing to individual ranges, a simple conclusion such as “IL-10 is high, while IL-2 is low” for some patient may be drawn, while a more complex algorithm may determine the abnormality of some sample based on less easy-to-interpret factors. One possible way to work around this is to use a combination of sophisticated but accurate algorithms, along with algorithms that yield outputs which are more easy to understand, such as COP.

Another possible drawback is that, as currently implemented, the algorithms determine anomalous profiles simply by finding profiles that are most ‘different’ to reference samples, while medically there may be no actual symptom or problem associated to some types of ‘different’ profiles. In other words, some anomalous profiles may be clinically significant, while some may not. Nonetheless, the intended use of such algorithms is to raise a flag that the cytokine profile is anomalous, and needs to be investigated further. Similarly, as currently implemented, all cytokines are treated equally by the algorithm, whereas in practice perhaps some cytokine imbalances are more significant than others, and so should possibly be given more weight by the algorithm. Having said that, “importance” may be difficult to quantify, and may also vary depending on the condition and context.

Finally, another drawback is that some of the algorithms which we used rely on a parameter *k* (the number of neighbours). Presently, there are no known precise methods for choosing optimal values for the parameter^15,19,26^ (unlike in classification problems, where hyper parameters can be tuned by cross-validation). Hence, we have used standard choices for the parameters (Methods) which have been found to be effective on a range of datasets and types. Nonetheless, the ROC AUC values reported here may be slight underestimates of the values which could be obtained with more optimally chosen parameters. It follows that if/when methods are developed to optimise the parameters of these algorithms, then we can expect them to be even more accurate and effective than we have found here.

In this work we have used the ROC AUC to measure outlier detection ability, and ROC AUC relies only on the outlier rankings of samples. In practice however, to declare a given sample anomalous or not, it is often useful to define a threshold such that all samples ranked in the top *x*% of most extreme samples are declared outliers, and all else inliers. Deciding on the value of *x* is context dependent, and will vary on the relative consequences of high false positive rates vs. high false negative rates. For example, a threshold of 10% might be used if ‘false alarms’ are acceptable and at the same time missing a true anomaly could have dire consequences. Alternatively, a threshold of 5% or 2.5% or 1% maybe also be appropriate, the latter especially if samples should only be flagged are anomalous if there is strong evidence to suggest this.

It is worth contrasting our work with a somewhat similar, but distinct method. It is common for researchers (e.g. ref.^10^) to use multivariate classification analysis to compare between samples of the medical complication with samples from normal/healthy individuals, and hence infer the typical cytokines which are elevated/depressed in certain conditions, i.e. the cytokine signature. However, these methods are not well suited for measuring how well a given *single* sample matches a cytokine signature. This is because classification algorithms are designed to determine which group a given sample is *more similar* to, but not necessarily *if* the sample is actually very similar to either group, or how similar. As a simple example, consider classifying a sample to group A or group B, where a binary classification algorithm has determined that biomarker values below 100 are classed as group A, and values above 100 as group B. Hence, if a new subject has a biomarker value of 200, then the sample will be classed as group B. This classification does not imply that the sample is in fact similar to samples in group B: If samples in group B typically range between 100 and 125, then 200 is very unusual, despite being classed as belonging to group B. While this was a simple univariate example, it can be seen that the analogous argument holds in the multivariate setting also. This problem is most pronounced for medical diagnostics: If we wished to examine a patient’s cytokine profile to see if it is abnormal in *any way* with respect to healthy patients, it would not be appropriate to use classification because *a priori* we may not know which disease signature to compare to.

Finally, while we have set our work in the context of cytokine studies, the arguments apply also to more general multivariate analyses with large numbers of biomarkers, such as the study of Nolen *et al*.^28^ where the values of 211 biomarkers from urine samples of healthy donors were derived. In this context, for example, it would be interesting to create a biomarker signature using anomaly detection for these data also, so that e.g. patients could easily compare their samples to these, and learn whether or not, and in which ways, their biomarker profile matches the other healthy samples. For future work, we hope that similar tools and methods as described here will be used to investigate larger and more diverse biomarker data sets, and help establish accurate, quantitative, and objective multivariate reference signatures for a range of groups and biomarkers.

## Methods

### Multivariate algorithms and parameters

Classical statistical methods for outlier detection (identification, rejection) are based on assumptions regarding the family of distributions of objects. Barnett and Lewis^29^ discuss in their classical textbook numerous tests for different distributions. The tests are tailored for each family of distribution and dependent, e.g., on the specific parameters of the corresponding distribution, the number of expected outliers, the number of variables, i.e., these are parametric methods. With an increasing number of variables and potential multi-modality, it becomes difficult or even impossible to apply these methods correctly. In such data, or in general when no information or reasonable guess concerning the nature of the data distribution are available, often non-parametric methods are therefore used^30^. Such methods compare local density estimates for the object in question with other local density estimates of neighbors, i.e. locally, or of all other objects. There is a broad variety of such methods, differing in the way density estimation is performed, how local models are built on top of the density estimate, or how local models are compared^31^. We experiment with several popular algorithms; a brief description of these follows now.

#### KNN

*k*-nearest neighbours (KNN) is a standard method for anomaly detection, which is based on the distance of each sample from its neighbouring data samples. Essentially, a sample which is atypically far from its neighbours is declared an anomaly. More precisely, two variants are popular: Either the actual distance to the *k*th closest data point is used to measure how far a sample point is from its neighbours, or the average distance to the the *k* closest data points is used. Here we use the former^20^. For multivariate data, Euclidean distance is used. Either way, this process assigns a real valued distance to each sample, which thereby acts as an outlier score — larger distances imply more outlying samples. These scores are then used to rank samples according to their outlying degree.

The parameter *k* determines how many close-by samples to count as a “neighbour”. We use a typical standard value for *k*, namely *k* = 20. For unsupervised outlier detection, optimally tuning *k* is an open problem. Nonetheless, using a value of around 20 can be seen to be mathematically reasonable because averaging distances over *k* = 20 samples is large enough such that the distance average will not be subject to overly dominant statistical fluctuations. On the other hand, 20 is typically small enough such that the distance is still a “local” estimate of density (assuming that the number of samples is *n* > 20, but ideally $n\gg 20$).

#### LOF

Local Outlier Factor (LOF)^19^ is a very popular method for anomaly detection, which is fundamentally quite similar to the *k*-nearest neighbours method. LOF employs estimates of the local data point density for each sample, and then assigns an outlier score to each sample which roughly corresponds to how much further a query point is to its *k*-nearest neighbours, compared to how far they are from each other. This aspect of employing the relative distance of a sample from its neighbours, rather than simply the absolute distance, is what sets it apart. Because of this local density aspect, LOF can handle locally varying data densities (which KNN typically cannot), and hence complex multivariate distributions (e.g. with strong skew). The relative distances act as the LOF outlier score. For example, a sample with a LOF score of ~1 means that the sample is relatively as close to its neighbours as they are from each other (hence the point is an inlier). On the other hand, if the LOF score is ~1, then the sample point is relatively much further from its neighbours than they are to each other, and hence the sample point will be declared an outlier. There is no universally agreed cut-off threshold to be declared an outlier, but usually LOF scores >3 would suffice. Alternatively, given a sample of data, the samples can be ranked by their LOF scores, and a threshold can be determined by the score which separates the the top 5% (say) most outlying points from the bottom 95%. We use a typical standard value for *k*^18,19^, namely *k* = 20. The same mathematical rationale applies for the choice of *k* as for the choice in the KNN method.

#### LoOP

Local Outlier Probability (LoOP)^18^ is a variant of LOF using a more robust local density estimate, and a normalization of the outlier scores. Outlier scores are thus given in the range [0,1], and the score for each point can be interpreted as the probability that a sample point is an outlier. Thus, samples in dense regions will have values ≈0 and samples in low density regions will have ≈1. We use a *k* = 20 again, because it is a standard choice^18^, and is mathematically reasonable, as discussed already.

#### ABOD

The Angle Based Outlier Detection (ABOD)^23^ method is founded on the intuition that an outlier in a multivariate data set will have, by sitting on the ‘edge’ or outside a single cluster of data samples, smaller variation in the angle formed when the sample under investigation forms a triangle by making two lines from itself to two other typical data samples in the cluster. Thus data points with smaller variances in their angles to other data points are more likely to be outliers. Mathematically, the ABOD score for sample *X* is obtained by first calculating the distance weighted cosine of the angle between vectors *Y* − *X* and *Z* − *X*, for all pairs of vectors *Y* and *Z* in the dataset (of course assuming *X* ≠ *Y*, *X* ≠ *Z*, *Z* ≠ *Y*). The variance in the cosine angle is the outlier score, with smaller variances implying more outlying samples. The ABOD score can be used to rank all the samples in a dataset by outlying degree. A positive aspect of this method is that there are no parameters to set. A negative aspect is that the method does make assumptions about the structure of the data, and type of outliers.

#### Isolation Forest

The Isolation Forest (IsolFor) method^22^ uses random trees similar to decision trees, and it rests on the intuitive observation that anomalous samples from a data set can usually be isolated by only a few variables and variable-threshold values. So, if a sample is anomalous due to a single variable having an extremely large value, then this sample can be isolated from the remaining data by just using this one variable and specifying one threshold value. In contrast, isolating a typical (i.e. non-anomalous) member of the data set will require specifying many variables and threshold values. Because such isolations can be represented as paths down a threshold-based decision tree, anomalous samples are those with atypically short paths to reach the sample. Many random trees are built by subsequently choosing random variables/features of the data, and branching at random cut-off thresholds for each variable, to make trees. Each random tree will give a different distance from the tree root to each samples node, and these distances are simply averaged. The length of the average path determines the outlier score, with shorter lengths being more outlying. As an example, consider an outlier which has large values of most of its variables. When the first variable is chosen for the tree, it is likely to be one in which the outlier has a large value. Further, when a random threshold is chosen in which to split the variable into two branches, it is likely that the outlying point is separated from the remaining data points by this random cut. Hence this outlier would already be isolated from the other sample, with a very short path length from the tree root.

Some advantages of this method are that it is suitable for a variety of distributions including strongly skewed variables, it has essentially no tuning parameters, data preprocessing is minimal, it is suitable for small data sets, and it is fast to compute. Further, it is simple to understand and the decision tree basis makes interpretation relatively straightforward. There are no parameters to set, except for the number of estimators, which we set to 10^3^, i.e. we have an ensemble of 10^3^ trees. As with any estimate of an average via sampling, the more samples used, the more accurate the estimate eill be. Hence in general using, a higher number of ensembles is better. Of course, a higher number of ensembles will increase the computational cost, so this aspect must be considered also. As a rule of thumb, increasing the number of ensembles until consistent outlier scores are obtained is one way to tune this parameter.

#### GLOSH

Global-Local Outlier Scores from Hierarchies (GLOSH)^21^ is a method for outlier detection that comes along with hierarchical density estimates provided by the hierarchical clustering method HDBSCAN*. Outliers are scored with respect to the closest cluster in the hierarchy, a measure that is, due to the hierarchical cluster structure, adaptive to a local or a global scale. The parameter defining the number of points for density estimates and the minimum cluster size is set to 3. The rationale for this value is that in our experiments we have quite small sample sizes, and so we must accept even very small clusters of points as being true clusters. With more samples, it may be possible to accept as true clusters only those clusters with at least 5 or 10 (say) samples, and any smaller ‘cluster’ of samples as mere outliers which happen to be in similar locations, and not a true cluster in the data.

#### COP

The method Correlation Outlier Probabilities (COP)^17^ is based on local neighborhoods in the spirit of LOF and LoOP, but uses principle component analysis (PCA) to derive a lower dimensional linear correlation structure in the neighbourhood. The worse the fit of an object to this local correlation, the higher the probability of being an outlier. Because PCA requires multiple data samples for each dimension, a relatively large neighbourhood is usually required for high-dimensional data. As a rule of thumb, the neighbourhood should exceed 3*d*^25^. Here we used *k* = 3*d* + 1 for the all data sets, which is chosen to satisfy 3*d* < *k* < *n*, and hence large enough for the PCA, but small enough to exclude some of the furthest data samples from the given sample under investigation. The condition 3*d* < *k* < *n* requires many more samples than the dimension of the data, and hence COP may not be applicable to some data sets with relatively few samples.

### Computational tools

The data mining software *Environment for Developing KDD-Applications Supported by Index-Structures* (ELKI)^32^ is used for implementations of KNN, LOF, LoOP, COP, and ABOD. *Sci-kit learn*^33^ is used for the implementation of Isolation Forest. GLOSH is implemented in Python by McInnes *et al*.^34^. Finally, *IPython*^35^ is used throughout this work.

### Handling missing values

The few missing values in the normal delivery and PIH data were handled using multiple imputation (with the number of multiple imputations set to 5), imputed using multivariate imputation by chained equations (MICE)^36^ (implemented using in fancyimpute in Python). The other data sets did not contain any missing values.

For the 5 imputations with the ND vs. PIH-out data, the mean ROC AUC values for the different algorithms (COP, LoOP, LOF, KNN, GLOSH, Isolation Forest, ABOD) were 0.87, 0.83, 0.94, 0.94, 0.79, 0.94 and 0.95, with standard deviations 0.03, 0.05, 0.01, 0.01, 0.09, 0.02 and 0.01 respectively. For the 5 imputations with the ND and PIH-in vs. PIH-out data, the mean ROC AUC values for the different algorithms were 0.83, 0.86, 0.94, 0.97, 0.80, 0.95, and 0.96, with standard deviations 0.03, 0.03, 0.01, 0.01, 0.10, 0.01 and 0.01 respectively. So there was relatively little variation between the different imputations.

### Data pre-processing

We logarithmically transform all the data, because a logarithm scale is more natural due to the order of magnitude variation in pg/ml cytokine concentration values. After this transformation, we scale the data in the standard way, by subtracting the mean and dividing by standard deviation. The outlier data was scaled by the mean and standard deviation of the inlier data, to ensure identical scaling. Scaling is important, because most of anomaly detection algorithms employed here mainly use distances between samples, hence different scales for different variables are undesirable.

### Subsampling the outlier group

In our data sets we will subsample the outlier group, so that when these subsamples are added to the inlier group ~5% of the combined samples are outliers. The subsamples are chosen uniformly from the outlier dataset, and outlier data points can appear at most once within each subsample. Outlier data points can appear in multiple subsamples however, because each subsample is independent of all others. For example, if the inlier dataset has *n* = 100 samples, and the outlier dataset has 200 samples, then multiple random subsamples of size 5 (=0.05*n*) are chosen from the 200 outlier samples. This is a standard method when calculating ROC AUC values, because the outliers must be rare in the combined data set. Clearly, if all outlier samples were added to the inlier samples, then the ‘outliers’ may in fact become very common, and hence not detectable as outliers. Each random subsample will yield an ROC AUC value, and we report the average ROC AUC values from 1000 random subsamples.

## Supplementary information

ROC curves

Supplementary Dataset 1

## Data Availability

The data for the normal and GBM example are available from ref.^37^. All other data sets are available as Supplementary Information to this article. All outlier algorithms are freely available for download - see Methods for references. Details of code used in this work are available from K.D. on request.
