# Supplementary material for: Establishing a many-cytokine signature via multivariate anomaly detection
Source: Sci Rep. 2019 Jul 4;9:9684. doi: 10.1038/s41598-019-46097-9 (PMC6609612; doi:10.1038/s41598-019-46097-9)
Supplement: Supplementary file 1 — ROC curves [file 41598_2019_46097_MOESM1_ESM.pdf]

Supplementary Information for:
Establishing a many-cytokine signature via multivariate anomaly detection

K. Dingle[1], A. Zimek[2], F. Azizieh[1] and A. R. Ansari[1]

[1]*International Centre for Applied Mathematics and Computational Bioengineering, Department of Mathematics and Natural Sciences, Gulf University for Science and Technology, P.O. Box 7207, Hawally 32093, Kuwait*

[2]*Department of Mathematics and Computer Science, University of Southern Denmark, Campusvej 55, 5230 Odense M, Denmark*

I. ROC FIGURES

Receiver operator characteristic (ROC) graphs display the classification false positive rate (FPR, the fraction of inliers which are declared outliers) vs. the true positive rate (TPR, the fraction of outliers which are declared outliers), for different values of a cut-off threshold, t. Any sample with outlier score above t (or below t, depending on the definition) is declared an "outlier", and an "inlier" otherwise. By varying t, higher and lower true and false positive rates can be found. If extreme values of t are used, then it is easy to make either the FPR or the TPR very high or low. For example, by choosing t very large, then almost all samples will be declared inliers, and hence lower the FPR to nearly 0%, but typically this will also lower the TPR, which is undesirable. However, it is non-trivial to lower the FPR *and* raise the TPR at the same time. Hence an algorithm is considered to have performed well if it can simultaneously achieve a low FPR and a high TPR. It follows that ROC curves which are very steep (i.e. achieve high TPR with low FPR), and which thereby have larger areas under them, indicate better performing classification. A common way to quantify and summarise the performance of algorithms is simply to quote the area under the ROC curve, known as receiver operator characteristic area under the curve (ROC AUC). We use the ROC AUC metric throughout this work. The ROC AUC also has a useful interpretation, which is as the probability that a randomly chosen inlier-outlier pair are correctly ranked by an algorithm as less and more outlying, respectively.

Here we provide some example ROC plots for each dataset used in this work. We use Isolation Forest for these example plots; see Figure 1. Also plotted (red dashes) is the expected ROC curve for a poor algorithm, which essentially guesses classifications at random, relying only on chance. Classification by chance would only correctly rank randomly chosen inlier-outlier pairs 50% of the time, and so such an algorithm would achieve an ROC AUC value of 0.5. Finally, the higher the ROC curve is above the chance line, the better the classification ability.

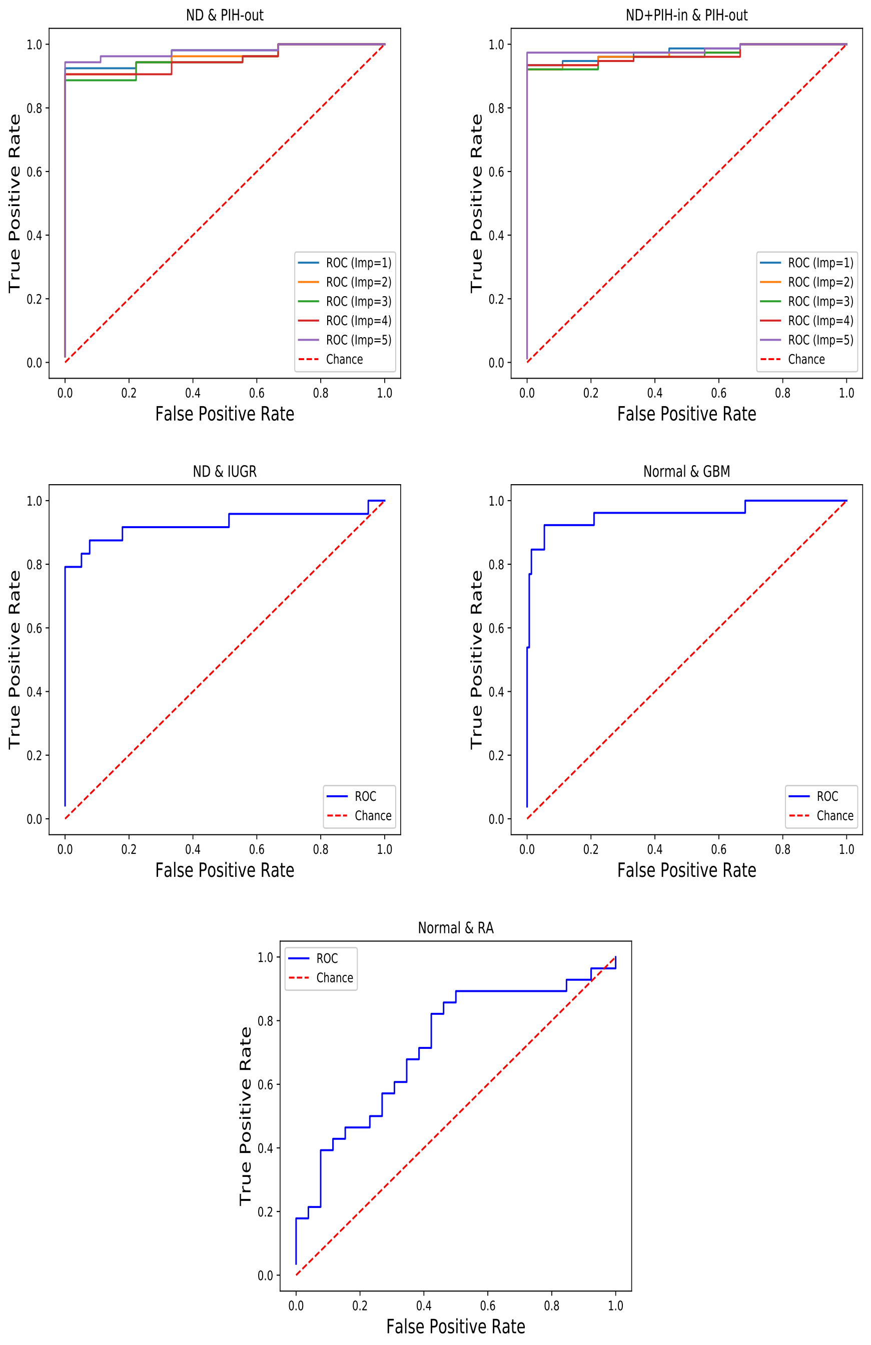

FIG. 1. ROC curves for the datasets examined in the main text. Panels including PIH data show multiple ROC curves, one for each of the imputations (see Methods). However, the differences between the ROC curves from multiple imputations are very small. Consistent with the analysis in the main text, all ROC curves have large areas and are steep, except for the normal vs. RA dataset which is less easy to classify.
